# The Evolving Role of Mesenchymal Stem Cells and Their Exosomes in Epilepsy Management: From Bench to Bedside

**DOI:** 10.1155/sci/4989846

**Published:** 2026-01-28

**Authors:** An Li, Zhuohui Zhao, Rulin Mi, Guofang Xue

**Affiliations:** ^1^ Department of Neurology, The Second Hospital of Shanxi Medical University, Taiyuan, 030001, Shanxi Province, China, sxmu.edu.cn

**Keywords:** epilepsy, exosomes, mesenchymal stem cells, neuroinflammation, treatment

## Abstract

Epilepsy affects over 70 million individuals globally, with nearly one‐third of patients failing to achieve seizure control despite the continued availability of new technologies and medications. Current epilepsy research aims to prevent or arrest the onset and progression of epilepsy by seeking novel therapeutic targets and developing potent medications. Neuroinflammatory pathways may underlie the core pathophysiology of epileptogenesis, according to evidence from clinical and fundamental research. Intervening in neuroinflammatory pathways can delay the onset and progression of epilepsy. Mesenchymal stem cells (MSCs) have recently garnered notable attention for their robust immunomodulatory and anti‐inflammatory properties in the context of inflammatory and immune‐mediated diseases, suggesting their potential as promising candidates in epilepsy management. The therapeutic efficacy of MSCs is largely ascribed to their paracrine function, particularly exosomes, as confirmed by numerous pertinent studies. This review synthesizes preclinical and clinical studies of MSCs and their exosomes in epilepsy treatment, elucidating their mechanisms of action. Collectively, these studies indicate that MSCs and their exosomes have the potential to serve as innovative epilepsy treatment in the future.

**Trial Registration:** ClinicalTrials.gov identifier: NCT05886205

## 1. Introduction

Epilepsy, a prevalent neurological disorder, impacts over 70 million individuals worldwide [1]. Currently, antiseizure medications represent the principal therapeutic approach for epilepsy. Nevertheless, despite adherence to optimal dosing regimens and rational medication use, a significant proportion—approximately one‐third—of patients still fail to achieve seizure control [2, 3]. The efficacy of current antiseizure interventions is limited, as they address the symptoms rather than the underlying causes and mechanisms of epilepsy, and they do not prevent or delay disease onset and progression [4–6]. The thrust of current research is directed towards the development of potent drugs and the discovery of innovative therapeutic targets aimed at preempting or arresting the onset and progression of epilepsy [7]. Epilepsy researchers have shifted their hot spot to neuroinflammatory targeted therapies since a significant number of studies have revealed the role of neuroinflammation in the pathophysiology of epilepsy, suggesting that the onset and progression of epileptic disorders can be delayed by intervening in neuroinflammatory pathways [8, 9].

Stem cells are characterized by their remarkable ability to self‐replicate and differentiate in multiple directions, enabling them to regenerate into a variety of tissues and organs. Stem cells can be categorized based on their differentiation potential into totipotent, subtotipotent, pluripotent, and monopotent stem cells. Mesenchymal stem cells (MSCs), a type of multipotent stem cell of mesodermal origin, can be derived from various sources, including bone marrow, umbilical cord, adipose tissue, dental pulp, and skin [10]. MSCs are among the few stem cell types that have been translated into clinical practice owing to their benefits, including accessible collection, minimal immune antigenicity, individualized treatment, and the absence of ethical controversy [11, 12]. MSCs are also recognized for their potent immunomodulatory and anti‐inflammatory properties, positioning them as a candidate treatment for neurological diseases associated with inflammation [13, 14]. In the context of epilepsy therapy, MSCs have recently garnered significant attention and are anticipated to serve as a novel therapeutic strategy of epilepsy in the future.

Historically, the therapeutic role of MSCs was thought to be mediated by their migration to damaged tissues and subsequent interaction with local cells following transplantation and infusion. However, a growing body of evidence from recent studies indicates that the primary therapeutic effects of MSCs are exerted through paracrine mechanisms, particularly via exosomes [14–18]. Exosomes are nanosized extracellular vesicles (~40–100 nm in diameter) that originate from the endosomal pathway. They are formed by the inward budding of the endosomal membrane, leading to the creation of multivesicular bodies (MVBs). These MVBs subsequently fuse with the plasma membrane, releasing the exosomes into the extracellular space. Compared to MSCs, MSC–derived exosomes (MSC‐Exos) not only possess potent immunomodulatory and anti‐inflammatory properties, but also exhibit an inherent capacity to penetrate the blood–brain barrier (BBB) and fewer adverse effects [15]. This review comprehensively examines the application of MSCs and their exosomes in epilepsy treatment, drawing conclusions on their mechanisms of action and therapeutic potential.

## 2. Epilepsy Pathophysiology: Targeting Neuroinflammation

**Figure 1:**
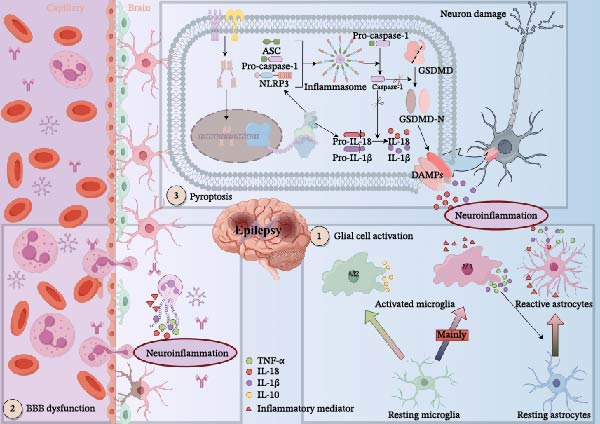
Pathophysiological mechanisms of epilepsy‐induced neuroinflammation. Following epileptic seizures, microglia become activated and predominantly polarize toward the pro‐inflammatory M1 phenotype. These activated M1 microglia release inflammatory mediators, triggering a pro‐inflammatory cascade. The secreted cytokines induce astrocytes to adopt a similarly pro‐inflammatory A1 state. This shift leads to the sustained release of pro‐inflammatory factors, thereby amplifying neuroinflammation. Concurrently, the breach of the blood–brain barrier allows peripheral immune cells and inflammatory mediators to infiltrate the brain, which exacerbates the neuroinflammatory response. Upon detecting danger signals, cells initiate inflammasome assembly and activate caspase‐1. Activated caspase‐1 then cleaves GSDMD, releasing its N‐terminal domain to form pores in the cell membrane. This results in pyroptosis—characterized by cell membrane rupture—and facilitates the release of cytokines, further intensifying the inflammatory cascade. The material in graphics by Figdraw.

Traditional antiseizure medications primarily exert their effects by modulating ion channels, yet they demonstrate limited efficacy in approximately one‐third of refractory epilepsy patients, suggesting additional mechanisms underpinning epileptogenesis [19]. In recent years, however, neuroinflammation has been recognized as a core pathological mechanism in epilepsy pathogenesis, with studies confirming that intervention in neuroinflammatory pathways can delay the onset and progression of epilepsy [20–24]. Epileptic seizures activate neuroinflammatory responses in the brain, manifesting as glial cell activation, BBB disruption, and programmed cell death, including pyroptosis [25, 26]. These responses further release substantial pro‐inflammatory cytokines, thereby creating a self‐perpetuating cycle that exacerbates neuronal hyperexcitability and disease progression [27–30]. The key pathophysiological mechanisms of epilepsy‐induced neuroinflammation are summarized in Figure 1.

In the pathophysiological mechanisms of epilepsy, glial cells play a pivotal role [30, 31]. As the resident innate immune cells of the central nervous system, microglia are the first to be activated following epileptic seizures, polarizing into a pro‐inflammatory M1 phenotype and releasing inflammatory mediators such as TNF‐α, thereby triggering an inflammatory cascade [32–34]. Concurrently, activated M1 microglia induce astrocytes to transition into a pro‐inflammatory A1 phenotype by secreting cytokines including IL‐1β and TNF‐α [35]. Although astrocyte activation occurs slightly later than that of microglia, their activated state is more persistent. They significantly amplify neuroinflammatory responses through the sustained production of pro‐inflammatory cytokines, chemokines, prostaglandins, and other mediators [36]. It should be noted that microglia may also undergo a shift towards the neuroprotective M2 phenotype, exerting antiepileptic effects through mechanisms such as clearing excess neurotransmitters, modulating synaptic activity, and secreting anti‐inflammatory factors [37].

BBB dysfunction, which is critical for maintaining central nervous system homeostasis, represents another core feature of neuroinflammation [38–40]. Since the seminal 1986 study by Cornford and Oldendorf [41], substantial evidence has shown that epilepsy disrupts BBB integrity. Clinical studies have detected elevated levels of BBB–associated proteins, such as MMP‐9, in both serum and cerebrospinal fluid of epileptic patients. Novel imaging techniques have also provided direct evidence of this damage [42–45]. This breach of the BBB allows immune cells and plasma proteins from the bloodstream to infiltrate the brain parenchyma, further amplifying the inflammatory response [46].

Pyroptosis, a pro‐inflammatory form of programmed cell death, is mediated by inflammasomes [47]. In the canonical pathway, inflammasome‐activated caspase‐1 cleaves the GSDMD protein. This cleavage not only promotes the maturation and release of IL‐1β and IL‐18 but also leads to pore formation and eventual rupture of the cell membrane, resulting in the massive release of inflammatory cytokines [48–50]. Upregulation of NLRP1/NLRP3 inflammasomes and their downstream components has been consistently observed in the hippocampal regions of both epilepsy patients and animal models [51–55]. The inflammatory cytokines released via pyroptosis enhance neuronal excitability and promote abnormal discharges, thereby establishing a vicious cycle between neuroinflammation and epileptic seizures [56]. Studies have confirmed that inhibiting the pyroptosis pathway reduces neuroinflammation and seizure severity while exerting neuroprotective effects, highlighting its potential as a novel and promising therapeutic target for epilepsy [54, 55, 57–61].

## 3. MSCs and Epilepsy

### 3.1. Therapeutic Mechanisms of MSCs in Epilepsy

MSCs are a type of multipotent stem cell originating from the mesoderm during early development, offering advantages such as abundant tissue availability, easy collection, low immune rejection, and minimal ethical controversy. MSCs can differentiate into not only various cell types of mesodermal origins like adipocytes, osteoblasts, and chondrocytes but also cells of ectodermal origins like neurons, astrocytes, oligodendrocytes, and cells of endodermal origin like hepatocytes [13, 62]. Chronic recurrent seizures can lead to neuronal apoptosis and necrosis. MSCs can restore the balance of the brain network after epilepsy by repairing damaged nerve cells and myelin sheaths, restructuring synapses and inhibiting the germination of aberrant mossy fibers, all facilitated by their robust regenerative capacity [63]. Moreover, MSCs may provide potential therapeutic benefits for structural causes of epilepsy, such as hippocampal sclerosis due to the above properties.

In 2017, the International League Against Epilepsy (ILAE) proposed six potential causes of epilepsy: genetic, structural, infectious, immune, metabolic, and unknown [64]. Infections and immune‐induced inflammatory processes can be both causal and consequential to seizures. MSCs have garnered extensive attention for their potent immunomodulatory and anti‐inflammatory capabilities in treating inflammatory and immune diseases [13]. MSCs produce and release substantial immunosuppressive factors, chemokines, and anti‐inflammatory cytokines as they migrate into the inflammation microenvironment [65, 66]. MSCs are anticipated to serve as a potential treatment for epilepsy by intervening in neuroinflammation. Preclinical and clinical research has already demonstrated their capacity to alleviate the severity of epilepsy and to mitigate excitotoxicity, oxidative stress, and neuroinflammation that are induced by epilepsy [67].

Additionally, adenosine is an endogenous brain activity inhibitor with neuroprotective and anticonvulsant properties [68, 69]. MSCs therapy, an emerging treatment for epilepsy, has been demonstrated to facilitate the reconstitution of the adenosine system, thereby achieving antiepileptic and neuroprotective effects [70, 71].

### 3.2. Preclinical Study of MSCs for the Treatment of Epilepsy

#### 3.2.1. Umbilical Cord MSCs

Huang et al. [72] evaluated the effects of intrahippocampal transplantation of human umbilical cord MSCs (hUC‐MSCs) in rats with pilocarpine‐induced epilepsy. Compared to the model group, the incidence and duration of spontaneous recurrent seizures in epileptic rats are reduced by hUC‐MSCs. In histopathology, hUC‐MSCs reduce neuron and interneuron loss, inhibit glial cell activation, and reduce abnormal mossy fiber sprouting. This study aimed to investigate the potential mechanisms of hUC‐MSCs by analyzing cytokine expression in hippocampal tissue using a human cytokine array. MSCs transplanted in the hippocampus released cytokines that exerted neuroprotective and anti‐inflammatory effects, such as FGF‐6, amphotericin, glucocorticoid‐induced tumor necrosis factor receptor (GITR), MIP‐3β, and osteoprotectin, as demonstrated by the results. The results of this study indicate that hUC‐MSCs may have a therapeutic effect in epilepsy models by releasing neuroprotective and anti‐inflammatory cytokines.

To compare the effectiveness of MSCs with antiepileptic drugs, Mohammed et al. [73] explored the therapeutic effects of hUC‐MSCs compared to gabapentin in pentylenetetrazole‐induced chronic epileptic rats. Compared to the model group, both hUC‐MSCs and gabapentin improved oxidative stress injury and motor and cognitive deficits in epileptic rats; however, the effect of hUC‐MSCs was more pronounced. The present study also discovered that MSCs may act by increasing the levels of GABA neurotransmitters.

(18) F‐fluorodeoxyglucose positron emission tomography (FDG‐PET) is a noninvasive technique for monitoring the body’s glucose metabolism. Park et al. [74] applied FDG‐PET to evaluate the efficacy of unilateral intrahippocampal transplantation of hUC‐MSCs in rats with chronic epilepsy. Despite the transplanted MSCs survived in the hippocampus, they failed to differentiate into specific neuronal cell types in the present study. The implanted MSCs can subsequently migrate to the contralateral hippocampus, partially restoring bilateral hippocampal glucose metabolism. Furthermore, the hippocampal glucose metabolism remained stable for at least 8 weeks after transplantation. The present study suggests that hUC‐MSCs may have a promising effect on epileptic networks.

#### 3.2.2. Bone Marrow MSCs (BMSCs)

Salem et al. [75] investigated the role of BMSCs in a rat model of pilocarpine‐induced epilepsy. In the present study, transplantation of BMSCs maintained average amino acid neurotransmitter levels, downregulated immunoreactivity to insulin growth factor‐1 receptor, synaptophysin, and cysteinyl asparagine‐3, and reduced oxidative damage and neuroinflammation in an epilepsy model. Despite the clinical advantages of intravenous injections, this study found that hippocampal injections increased the number of cells in intracranial BMSCs compared to intravenous injections.

In an additional study, Fukumura et al. [76] found that intravenous injection of BMSCs reduced seizures’ frequency and improved cognitive function in epileptic rats. The study further demonstrated that MSCs exert neuroprotective effects by reducing neuronal cell death and inhibiting abnormal mossy fiber sprouting.

Adenosine receptors are a class of G protein‐coupled receptors with four subtypes that bind to adenosine to exert biological effects [77, 78]. The activation of adenosine A1 receptors is anticonvulsant, whereas the activation of A2A receptors may trigger epilepsy [79]. Huicong et al. [70] studied the dynamics of adenosine A1 and A2a receptors in an epilepsy kindling model. The findings indicated that the expression of A1 receptors in the rats’ hippocampus increased in the epilepsy kindling model at 24 h and gradually decreased at 1 and 6 months. In contrast to A1 receptors, the expression of A2a receptors exhibited an opposing trend in this study. This research substantiates the ability of MSCs to rectify imbalances in the expression of the adenosine receptor in epilepsy models. Kang et al. [71] conducted an additional study in which they transplanted BMSCs into the CA3 region of the hippocampus of epileptic rats. The results indicated that the hippocampus of rats showed an increase in adenosine A1 receptor expression and a decrease in A2a receptor expression at 3 months following MSCs transplantation and significantly improved epileptiform discharges.

#### 3.2.3. Adipose MSCs (ADSCs)

Wang et al. [80] explored the impact of ADSCs on a model of epilepsy induced by kainic acid. Compared to controls, ADSCs transplantation reduces seizure frequency and enhanced learning and memory functions in epileptic rats. Additionally, it was discovered that ADSCs mitigate hippocampal apoptosis by releasing neuroprotective factors such as BDNF, NT3, and NT4. Similarly, Waloschková et al. [81] investigated ADSCs in a comparable model and confirmed these therapeutic benefits. Their study also demonstrated that ADSC transplantation reduced seizure frequency, improved short‐term memory, and additionally alleviated anxiety in epileptic rats.

#### 3.2.4. Other MSCs

Olfactory mucosal MSCs (OM‐MSCs) are considered the clinically preferred source of MSCs due to their safety and accessibility. Liu et al. [82] investigated the effects of OM‐MSCs on pilocarpine‐induced epilepsy in mice. In comparison to controls, OM‐MSCs transplantation reduces seizure frequency and improves cognitive, motor, and perceptual functions in epileptic mice following OM‐MSCs transplantation. The investigation also discovered that transplanted OM‐MSCs may recruit Treg cells to suppress inflammation and reconstruct neural networks.

Senthilkumar et al. [83] systematically compared the therapeutic effects of dental pulp stem cells (DPSCs) and BMSCs in a kainic acid induced epilepsy mouse model. The findings revealed that intravenous infusion of both stem cell types effectively alleviated hippocampal neurodegeneration and neuroinflammation and significantly improved epilepsy‐associated neuropsychiatric comorbidities, such as cognitive impairment, hyperactivity, and depressive‐like behavior. Notably, the therapeutic efficacy of DPSCs was comparable to that of BM‐MSCs across most parameters. Furthermore, the study observed negligible engraftment of the transplanted cells in the brain, strongly suggesting that the therapeutic benefits are primarily mediated by paracrine mechanisms rather than direct cell replacement or long‐term engraftment.

The summary of preclinical studies of MSCs therapy for epilepsy is presented in Table 1.

**Table 1:** Preclinical studies of MSCs therapy for epilepsy.

Author	MSCs source	Dose	Injection route	Model of epilepsy	Main histopathological findings	Main clinical findings
Huang et al. [72]	hUC‐MSCs	1 × 10^5^ cells	Intrahippocampal transplantation	Pilocarpine TLE model of rats	Reduce brain edema and hippocampal cytoarchitecture changes; decrease of neuron and interneuron loss, inhibited astrocyte and microglial cells activation, and a reduction of mossy fiber sprouting; release growth promoting factor	Suppress the incidence and duration of spontaneous recurrent seizures
Mohammed et al. [73]	hUC‐MSCs	1 × 10^6^ cells	Tail vein injection	Pentylenetetrazole chronic epilepsy model of rats	Enhance the GABA neurotransmitter levels oxidative; improve stress damage	Improve motor and cognitive impairments
Park et al. [74]	hUC‐MSCs	5 × 10^5^ cells	Intrahippocampal transplantation	Pilocarpine‐induced SE model of rats	Increase glucose metabolism in the hippocampus	Seizure frequency not different
Salem et al. [75]	BMSCs	1 × 10^5^ cells or 3 × 10^6^ cells	Bilateral intrahippocampal injection or vein injection	Pilocarpine‐induced TLE model of rats	Ameliorate the neurochemical and histological changes; reduce the oxidative insult and inflammatory markers; retain amino acid neurotransmitters to the normal level; downregulate the immunoreactivity to insulin growth factor‐1 receptor, synaptophysin, and caspase‐3	IC‐administered showed more pronounced effects than those administered via IV route
Fukumura et al. [76]	BMSCs	1 × 10^6^ cells	Tail vein injection	Pilocarpine‐induced SE model of rats	Inhibit neuronal cell death and suppress aberrant MFS	Inhibit seizure frequency and improve cognitive function
Huicong et al. [70]	BMSCs	4 × 10^3^ cells	CA3 area of the right intrahippocampal injection	Lithium‐pilocarpine‐induced SE model of rats	Improv the dysregulated ARs expression	Reduce the amplitude and frequency of EEG spike waves
Kang et al. [71]	BMSCs	4 × 10^3^ cells	CA3 area of the right intrahippocampal injection	Lithium‐pilocarpine‐induced SE model of rats	Improve the imbalance in the A1 adenosine receptor/A2a adenosine receptor ratio	Improve epileptiform discharge
Wang et al. [80]	ADSCs	5 × 10^4^ cells	Left intrahippocampal injection	Kainic acid–induced epilepsy model of rats	Releases antiapoptotic BDNF, NT3 and NT4	Improve the learning and memory function; inhibit seizure frequency
Waloschková et al. [81]	ADSCs	6 × 10^2^ cells	Bilateral intrahippocampal injections	Kainic acid–induced epilepsy model of rats	Failure to suppress microglial activation	Reduce seizure frequency; improve short‐term memory and reduce anxiety
Liu et al. [82]	OM‐MSCs	1× 10^5^ cells	Intrahippocampal injection	Pilocarpine‐induced epilepsy model of mice	Recruit Treg cells to the brain, inhibit inflammation, rebuild the neural network	Improve the cognitive, locomotive, and perceptive functions
Senthilkumar et al. [83]	DPSCs or BMSCs	1 × 10^6^ cells	Tail vein injection	Kainic acid–induced epilepsy model of mice	Confer hippocampal neuroprotection; reduce neuroinflammation	Attenuate neuropsychiatric comorbidities like cognitive impairment, hyperactivity, impaired emotional responsiveness, and depression

### 3.3. Clinical Study of MSCs for the Treatment of Epilepsy

#### 3.3.1. BMSCs

In 2017, Hlebokazov et al. [84] conducted a Phase 1 clinical study that was single‐center, randomized, and open‐label. This study evaluated the safety and efficacy of autologous BMSCs for treating drug‐resistant symptomatic epilepsy. A total of 22 individuals were enrolled in the study, out of which 12 were included in the control group to receive standard treatment with antiepileptic. In contrast, the remaining 10 were assigned to the study group to receive antiepileptic drugs in combination with autologous MSCs therapy. Regarding efficacy, three of the 10 patients in the experimental group obtained remission, which is defined as being seizure‐free for 1 year or more, whereas only two of the 12 patients in the control group achieved remission. Regarding efficacy, MSCs injections were well‐tolerated and did not cause any severe adverse effects.

Based on their previous study, Hlebokazov et al. [85] conducted a more extensive Phase I/II open‐label clinical study in 2021. A total of 67 patients were included in the study, 33 in the control group and 34 in the experimental group. In terms of efficacy, the frequency of seizures was reduced by over 50% in 61.7% of patients in the experimental group at the 6‐month time point, compared to 24.2% in the control group. A second course of treatment was administered to 14 patients in the experimental group after the first course of treatment. The proportion of the experimental group with a reduction in seizure frequency of more than 50% increased to 76.5% at the 12‐month time point, in contrast to 3.0% in the control group. The HADS and MMSE scores of epileptic patients in the experimental group improved as compared to the control group. This study confirmed that recurrent MSCs therapy further reduces the number of seizures and epileptiform brain activity, suggesting that it may provide additional clinical benefits. Furthermore, the study determined that MSCs were more effective when combined with levetiracetam. Regarding safety, MSCs therapy did not cause any severe adverse effects.

In 2018, Milczarek et al. [86] evaluated the safety, feasibility, and potential efficacy of autologous bone marrow nucleated cells (BMNCs) combined with BMSCs for treating drug‐resistant epilepsy in children. In terms of safety, four children received the treatment without experiencing any adverse events during the 2‐year follow‐up period. In terms of efficacy, the number of seizures in the children was reduced from 10 per day to 1 per week, with the number of status epilepticus (SE) reduced from 4 per week to no more seizures. In addition, the therapy improved the children’s cognitive function and reduced the number of discharges in the EEG evaluation.

#### 3.3.2. Other MSCs

A 26‐year‐old woman was enrolled in a clinical study of OM‐MSCs for refractory epilepsy [82]. On April 22, 2014, and April 28, 2014, the patient respectively underwent two transplants of autologous OM‐MSCs by intrathecal injection. During the 8‐year follow‐up period, the patient’s seizure type transitioned from generalized tonic‐clonic seizures to absence seizures, and the frequency of absence seizures gradually decreased. Cranial magnetic resonance imaging (MRI) indicated that diffusion brain atrophy could be significantly reversed by the fourth year following treatment. No obvious adverse reactions or complications were observed during the 8‐year follow‐up in terms of safety.

The summary of Clinical studies of MSCs therapy for epilepsy is presented in Table 2.

**Table 2:** Clinical studies of MSCs therapy for epilepsy.

Author	Year and country	Study design	MSCs source	Injection route	Subject recruitment	Control group (number of subjects)	Therapy group (number of subjects)	Efficacy	Safety	Clinical trials ID
Hlebokazov et al. [84]	2017 Belarus	A single‐center Phase I open label study	Autologous BMSCs	Intravenous and intrathecal injection	Refractory epilepsy	Conventional ASMs (12)	ASMs supplemented by MSCs (10)	Improve seizure frequency and severity; improve cognitive and anxiety	Not exhibit any severe adverse reactions	NCT02497443
Milczarek et al. [86]	2018 Poland	A prospective, longitudinal experiment	Autologous BMNCs and BMSCs	Intravenous and intrathecal injection	Refractory epilepsy	No	Four children	Decrease the epileptiform discharges and significant reductions; reduce the epileptic seizures and SE episodes; ameliorate neurological and neuropsychological symptoms	Not exhibit any severe adverse reactions	Unregistered
Hlebokazov et al. [85]	2021 Belarus	A single‐center Phase I/II open‐label study	Autologous BMSCs	Intravenous and intrathecal injection	Refractory epilepsy	Conventional ASMs (33)	ASMs supplemented by MSCs (34)	Reduce the seizure count and epileptiform EEG activity; improve cognitive and anxiety	Not exhibit any severe adverse reactions	NCT02497443
Liu et al. [82]	2023 China	A prospective experiment	OM‐MSCs	Intrathecal injection	Refractory epilepsy	No	A 26‐year‐old female	Alleviate brain atrophy; improve seizure symptoms	Not exhibit any severe adverse reactions	ChiCTR2200055357

### 3.4. MSC Modification Therapy

MSCs are a promising therapeutic modality that has attracted significant attention for their clinical potential [87, 88]. However, their therapeutic efficacy in vivo is hampered by several limitations, including poor survival rates posttransplantation, insufficient homing to lesion sites, and an inability to sustainably release therapeutic factors at target locations [89]. To overcome these challenges, two main strategies are being employed: genetic engineering and in vitro preconditioning, both aimed at functionally enhancing MSCs to improve their therapeutic outcomes [90].

Genetic engineering techniques, such as gene editing or viral vector transduction, enable precise modification of MSCs to enhance their therapeutic functions [90, 91]. These strategies vary depending on the therapeutic goal. For instance, to augment paracrine signaling, MSCs can be engineered to overexpress anti‐inflammatory cytokines, neurotrophic factors, or specific miRNAs, thereby strengthening their capacity to modulate neuroinflammation and promote neurorepair [92]. Ali et al. [93] explored this approach in an epileptic mouse model using MSCs engineered to sustain secretion of IL‐13. Alternatively, suppressing undesirable gene expression via RNA interference is equally effective; Long et al. [94], for example, significantly reduced mortality and abnormal EEG activity in epileptic animals by inhibiting the Hes1 gene. Furthermore, overexpressing chemokine receptors on MSC surfaces can enhance their homing and accumulation at brain injury sites [95]. Concurrently, introducing antiapoptotic genes can improve cell survival within the harsh microenvironment of pathological lesions.

Beyond genetic engineering, in vitro preconditioning of MSCs prior to transplantation offers a practical and flexible strategy for functional enhancement. For instance, hypoxia preconditioning mimics the ischemic microenvironment of target lesions, which not only improves therapeutic efficacy but also upregulates antiapoptotic genes to enhance posttransplant survival [96, 97]. Cytokine pretreatment can activate the immunomodulatory functions of MSCs, boosting their secretion of anti‐inflammatory factors and overall regulatory capacity [98]. Similarly, pharmacological pretreatment may be used to selectively augment specific therapeutic properties of MSCs [99]. In summary, both genetic engineering and systematic in vitro preconditioning can significantly improve the therapeutic potential of MSCs for treating epilepsy and other neurological disorders.

### 3.5. Limitations of MSCs

Despite their significant therapeutic potential in immune and inflammatory diseases, the clinical translation of MSCs is constrained by several concerns, most notably regarding their safety profile [100]. First, their potent immunomodulatory properties may inadvertently suppress antitumor immune responses, potentially promoting tumor growth [101, 102]. Second, their capacity to differentiate into vascular endothelial cells and secrete pro‐angiogenic factors could exacerbate tumor metastasis by promoting angiogenesis [103–105]. Finally, while their multipotent differentiation holds regenerative promise, it also carries the risk of off‐target differentiation into unintended tissues such as bone or cartilage [106].

Regarding their biological characteristics, MSCs present several inherent limitations. First, they exhibit considerable heterogeneity; cells from different sources or donors vary in proliferation capacity, differentiation potential, and immunomodulatory function, which affects the consistency of therapeutic outcomes [107]. A more critical challenge is their inefficient delivery to target sites. Following systemic administration, most MSCs are sequestered by filter organs like the lungs, spleen, and liver, resulting in very low engraftment rates within the intended tissues [108]. Furthermore, therapeutic efficacy is constrained by the limited survival of transplanted cells. Most undergo rapid death or clearance, meaning their benefits rely primarily on transient paracrine actions rather than long‐term engraftment.

The clinical translation of MSCs is impeded by a series of challenges [109]. First, scalable and consistent manufacturing is not yet assured. Issues with cell expansion, storage stability, and batch‐to‐batch variability limit large‐scale production, compounded by a lack of unified regulatory and quality control standards [110, 111]. Second, the clinical evidence base is weak. Existing studies are often hampered by small sample sizes, insufficient follow‐up, and heterogeneous protocols, precluding robust comparisons and the development of clear treatment guidelines [112]. Finally, a limited understanding of the precise mechanisms by which MSCs exert their immunomodulatory and regenerative effects hinders the rational design and optimization of therapies [113]. Together, these gaps significantly slow the progression of MSCs from basic research to routine clinical implementation.

## 4. Exosomes—Mediators of MSC Action

### 4.1. Exosome Definition

Exosomes are a class of nanoscale (diameter 40–100 nm) extracellular vesicles, which are small vesicles released outside the cell after intracellular MVBs fuse with the cell membrane [114]. Almost all cell types, including tumor cells, can secrete exosomes, which carry biologically active substances such as nucleic acids, proteins, and lipids [115, 116]. The advantages of exosomes, an endogenous substance with excellent pharmacokinetics, unique immunological properties, and natural penetration of physiological barriers, have garnered attention in treating CNS disorders.

### 4.2. MSC‐Exos

Historically, MSCs were thought to exert their effects by migrating to sites of injury posttransplantation or infusion, interacting with surrounding cells. MSCs fulfill their paracrine function by secreting soluble factors or extracellular vesicles, and the role of exosomes in the secreted extracellular vesicles is especially significant [14–18]. MSC‐Exos contain a rich cargo of miRNAs, mRNAs, and proteins sourced from MSCs, which can be transferred to target cells to elicit anti‐inflammatory responses [18]. MSC‐Exos possess not only the powerful immunomodulatory and anti‐inflammatory properties of MSCs but also the exosomes’ ability to naturally cross the BBB, making them particularly advantageous in diagnosing and treating CNS diseases. Furthermore, MSC‐Exos is more convenient to store and administer than MSCs. MSC‐Exos have a low immunogenicity, substantially reducing the risk of malignant transformation despite the potential for malignant transformation with MSCs therapy [117].

### 4.3. Exosomes as Carriers of Brain‐Targeted Drugs

While cell therapies face delivery challenges, extracellular vesicles—particularly exosomes secreted by MSCs—have emerged as a promising alternative drug delivery system. Their inherent nanoscale dimensions, low immunogenicity, and excellent biocompatibility make them attractive candidates [118–120]. Research indicates that exosomes can carry small molecules or nucleic acid therapeutics across the circulatory system to brain tissue, where they exert therapeutic effects [121, 122]. Membrane proteins on exosomes may further facilitate directed migration toward target cells, potentially enhancing local drug concentration and reducing systemic side effects [123].

However, natural exosomes have limited targeting efficacy, with most being sequestered by the liver and spleen after intravenous administration, which restricts brain delivery [124]. To overcome this, engineered modification strategies are widely adopted to enhance BBB traversal and targeting [125]. Although MSC‐Exos possess some inherent homing capability, surface modifications can refine specificity. For example, fusing target‐specific ligands (e.g., rabies virus glycoprotein peptides) to exosomal membrane proteins enables targeting of cerebral vascular endothelial cells [126]; genetic engineering can also be used to express natural targeting peptides [127]. Additionally, chemical modifications such as conjugating superparamagnetic iron oxide nanoparticles can introduce magnetic guidance capabilities [128].

Beyond targeting, efficient drug loading is paramount. Active loading methods—including electroporation, ultrasonication, calcium chloride treatment, or freeze–thaw cycles—are used to encapsulate nucleic acids, proteins, or small‐molecule drugs into isolated exosomes [129, 130]. For hydrophobic drugs, passive loading via simple incubation or microfluidics‐assisted techniques can be employed [131, 132].

Despite representing a breakthrough in nanomedicine, engineered exosomes still face challenges in storage stability, scalable production, drug loading efficiency, and clinical translation [133]. Continued advancements in this field hold promise for establishing exosome‐based targeted therapies as a significant contribution to the treatment of human diseases.

## 5. Exosomes and Epilepsy

### 5.1. Therapeutic Mechanisms of Exosomes in Epilepsy

Even though there are still few studies on MSC‐Exos for treating epilepsy currently, MSC‐Exos have a mechanism of action similar to MSCs theoretically [134]. Neuroinflammation and neuronal cell death are diminished by exosomes enriched with anti‐inflammatory molecules and neurotrophic factors secreted by MSCs [135]. Existing preclinical studies have demonstrated that MSC‐Exos alleviate epilepsy‐induced neuroinflammation, oxidative stress, and neuronal damage and improve cognitive and memory deficits [136–138].

### 5.2. Preclinical Study of Exosomes in the Treatment of Epilepsy

Luo et al. [136] explored the effects of umbilical cord MSC–derived extracellular vesicles (MSC‐EVs) on pilocarpine‐induced epilepsy in mice. It was found that MSC‐EVs could attenuate oxidative stress and restore the structure of hippocampal neurons and mitochondrial dysfunction. In addition, this study found that Nrf2, a mediator associated with neuroinflammation and oxidative stress, is involved in the antioxidant role of MSC‐EVs in epilepsy. Similarly, Xian et al. [137] discovered that Nrf2 was involved in umbilical cord MSC‐Exos’ antioxidant and anti‐inflammatory effects in epileptic mice. Exosomes derived from umbilical cord MSCs improved hippocampal reactive astrocyte proliferation, inflammatory responses, and learning and memory impairment in epileptic mice by inhibiting Nrf2.

Long et al. [138] explored the role of marrow MSC‐EVs (A1 exosomes) in pilocarpine‐induced models of persistent SE in mice by nasal administration. The findings indicated that A1 exosomes reduced hippocampal glutamatergic and GABAergic neuronal damage and significantly attenuated hippocampal inflammation in the early phases after SE. In the chronic phase after SE, A1 exosomes repress neurodegeneration, neuroinflammation and aberrant neurogenesis while also restoring cognitive and memory impairment. The results also suggest that nasal administration of A1‐exosomes may be therapeutic in other neuroinflammation‐related neurological disorders.

The summary of preclinical studies of MSC‐EVs therapy for epilepsy is presented in Table 3.

**Table 3:** Preclinical studies of MSC‐EVs therapy for epilepsy.

Author	MSC‐EVs source	Dose	Injection route	Model of epilepsy	Main histopathological findings	Main clinical findings
Luo et al. [136]	hUC‐MSCs	50 μg	Tail vein injection	Pilocarpine‐induced SE model of mice	Alleviate oxidative stress; restore neuronal morphology alterations and mitochondrial dysfunction	Ameliorate cognitive decline
Xian et al. [137]	hUC‐MSCs	30 μg	Intraventricular injection	Pilocarpine‐induced SE model of mice	Reduce the hippocampal reactive astrogliosis; attenuates hippocampal inflammatory response	Restore learning and memory impairment
Long et al. [138]	BMSCs	30 μg	Intranasal administration	Pilocarpine‐induced SE model of mice	Repress neurodegeneration, neuroinflammation and aberrant neurogenesis	Restore cognitive and memory impairment

### 5.3. Clinical Study of Exosomes in the Treatment of Epilepsy

No clinical trials have yet been conducted using MSC‐Exos in epilepsy. The only registered clinical trial in this field led by Peking Union Medical College Hospital in China, involves induced pluripotent stem cell–derived exosomes (iPSC‐Exos). This single center and open‐label study, which commenced in June 2023, aims to evaluate the safety, tolerability, and preliminary efficacy of iPSC‐Exos nasal drops in patients with focal refractory epilepsy. The clinical trial will enroll 34 patients, divided into four groups based on the dose administered. Each patient will receive treatment via nasal delivery twice daily for 12 weeks. Adverse effects, seizure frequency, electroencephalogram, and MRI were assessed at 1–24 Week after exosome treatment. The clinical application of exosomes to epilepsy is currently in the early stages of investigation. Additional well‐designed clinical trials will be required to expedite their transformation to the clinic.

### 5.4. Route of Delivery of Exosomes

Exosomes are administered in multiple ways; the modes of administration of exosomes can be broadly classified into systemic and local administration [15]. Intravenous administration is the most widely used route of administering exosomes; in addition, nasal administration, intraperitoneal injection, ventricular injection, and oral administration have also been tried in animal models [139]. Researchers have studied the function of exosomes in epilepsy models by administering them intravenously, nasally, and ventricularly. Kodali et al. [140] administered extracellular vesicles derived from BMSCs for the KA‐induced epilepsy model by nasal administration. It was found that MSC‐Exos were effectively delivered to neurons and microglia in numerous regions of the bilateral forebrain following unilateral nasal administration, and neurons in brain injury regions exhibited a higher uptake rate.

### 5.5. The Role of Exosomes in the Diagnosis of Epilepsy

Exosomes are secreted by nearly all cells in both physiological and pathological conditions. Exosomes carry host cell–derived nucleic acids, proteins, lipids, and other biologically active substances that respond to host cell pathophysiological condition [124, 141]. Due to the BBB, there is the absence of a specific biomarker to objectively respond to the pathophysiological condition of the CNS [142]. After crossing the BBB, exosomes are released from brain tissues and enter blood, urine, and other body fluids. This technology has the advantages of being non‐invasive and easy to obtain, so it can potentially applicable to diagnosing central nervous system diseases [143].

The expression of biologically active substances in exosomes is altered in pathological states, which suggests that the expression of miRNAs, proteins, and other substances in exosomes can be analyzed to aid in diagnosing disease [144, 145]. The potential of miRNAs in exosomes for biomarker research has been demonstrated [146, 147]. Yan et al. [148] explored the differential expression of miRNAs in exosomes of patients with medial temporal lobe epilepsy with hippocampal sclerosis (mTLE‐HS). The findings showed that 50 microRNAs were differentially expressed in mTLE‐HS patients compared to controls. Further studies found that miR‐8071 exhibited the highest diagnostic value for mTLE‐HS and was correlated with seizure severity. Raoof et al. [149] similarly found that the expression of miR‐328‐3p was upregulated in plasma exosomes from patients with temporal lobe epilepsy. The above studies indicate that the expression of miRNA in exosomes is crucial for the diagnosis of epilepsy and the prediction of prognosis.

In addition, Lin et al. [150] analyzed the expression of proteins in serum exosomes from epileptic patients and healthy populations. The results showed that 76 proteins were differentially expressed in patients with epilepsy compared to the healthy population, of which six proteins were upregulated, and 70 proteins were downregulated. Further expansion of the clinical samples revealed particularly marked differences in the expression of coagulation factor IX (F9) and platelet reactive protein‐1 (TSP‐1). The study suggests that proteins in exosomes could serve as potential biomarkers for the diagnosis of epilepsy.

### 5.6. Exosome Modification Therapy

Exosomes are considered natural drug delivery carriers because of their high biocompatibility, targeting properties, and stability in the blood circulation [151]. Artificial modification of exosomes has attracted much attention in recent years, and the therapeutic effects of exosomes can be enhanced by modifying the exosome surface and delivering therapeutic cargoes [125]. Liu et al. [152] explored the function of MSC‐Exos loaded with miR‐129‐5p in a mice model of epilepsy. The results indicated that MSC‐Exos overexpressing miR‐129‐5p attenuated neuroinflammation and hippocampal neuronal damage. With advances in science and experimental techniques, it is feasible to target damaged brain cells by customizing exosomes carrying therapeutic molecules, thereby enhancing the therapeutic efficiency of exosomes.

## 6. Conclusion

**Figure 2:**
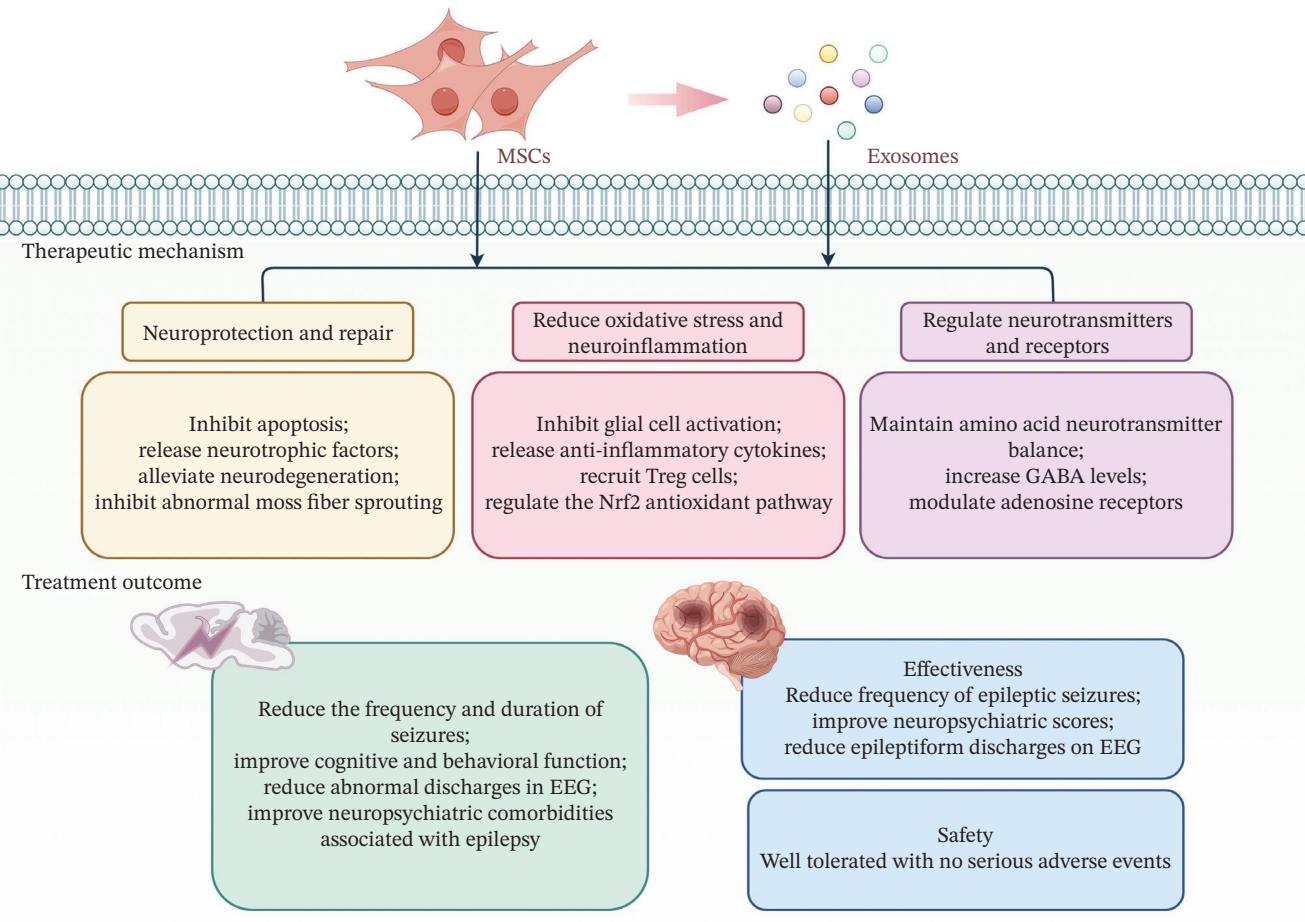
Mechanisms and therapeutic effects of MSCs and their exosomes in epilepsy. MSCs and exosomes exert antiepileptic effects primarily through three mechanisms: neuroprotection and repair, reduction of oxidative stress and neuroinflammation, and regulation of neurotransmitters and receptors. Preclinically, they reduce seizure frequency and duration, improve cognitive and behavioral function, and normalize EEG activity. Clinically, treatment lowers seizure frequency, improves neuropsychiatric scores, and demonstrates a favorable safety and tolerability profile. The material in graphics by Figdraw.

This review emphasizes the pivotal role of neuroinflammation in the pathogenesis of epilepsy and identifies MSCs and their derived exosomes as emerging therapeutic candidates. Their potential lies in multifaceted interventions that target the vicious cycle between neuroinflammation and neuronal hyperexcitability. A growing body of preclinical and clinical evidence suggests that MSCs and their derived exosomes can delay the onset and progression of epilepsy through mechanisms such as neuroprotection and repair, reduction of oxidative stress and neuroinflammation, and regulation of neurotransmitters and receptors (Figure 2). Moreover, MSC‐Exos offer significant advantages over MSCs: they can innately cross the BBB, exhibit lower immunogenicity and reduced tumorigenicity risk, and are more convenient to store and administer. Although clinical trials for MSC‐Exos in epilepsy treatment are ongoing, the initiation of the first registered trial marks a significant milestone in translating exosome therapy to the clinic.

Despite encouraging research findings, MSCs –based epilepsy therapies face several critical challenges in clinical translation. The current lack of large‐scale human clinical trials leaves uncertainties regarding optimal delivery routes, dosage regimens, and long‐term safety profiles. Furthermore, standardization issues in exosome isolation and characterization, alongside limitations in quality control, constitute significant translational barriers. Future research should prioritize the following strategic directions: advancing targeted therapies through engineered exosomes for enhanced precision; developing synergistic strategies that combine MSC‐Exos with conventional antiepileptic drugs; establishing exosome‐based biomarker platforms for early diagnosis and disease monitoring. Concurrently, critical research priorities remain, including addressing the heterogeneity of MSCs and exosomes, optimizing scalable production and standardized processes, and elucidating their precise molecular mechanisms. In summary, while MSC–based therapeutic strategies—particularly cell‐free exosome therapies—offer innovative pathways to modulate epilepsy progression, overcoming these translational hurdles is essential to fully realizing their potential.

## Author Contributions

All authors contributed to the conception and idea of the work.

## Funding

This work was funded by the Shanxi Province Higher Education “Billion Project” Science and Technology Guidance Project and MOE Key Laboratory of Coal Environmental Pathogenicity and Prevention (Grant BY‐ZB‐2024012), the Shanxi Provincial Science and Technology Department (Grant 202303021211213), and the Shanxi Province Postgraduate Practical Innovation Project (Grant 2023SJ147).

## Disclosure

All authors have critically reviewed drafts of the manuscript and approved the final version.

## Ethics Statement

The authors have nothing to report.

## Consent

The authors have nothing to report.

## Conflicts of Interest

The author declares no conflicts of interest.

## Data Availability

Data sharing is not applicable to this article as no new data were created or analyzed in this study.
